# Supplementary material for: A Cross-Sectional Survey of the Knowledge, Attitudes, and Practices of Antimicrobial Users and Providers in an Area of High-Density Livestock-Human Population in Western Kenya
Source: Front Vet Sci. 2021 Sep 21;8:727365. doi: 10.3389/fvets.2021.727365 (PMC8490823; doi:10.3389/fvets.2021.727365)
Supplement: Supplementary file 2 [file Presentation_1.PPTX]

## Slide 1
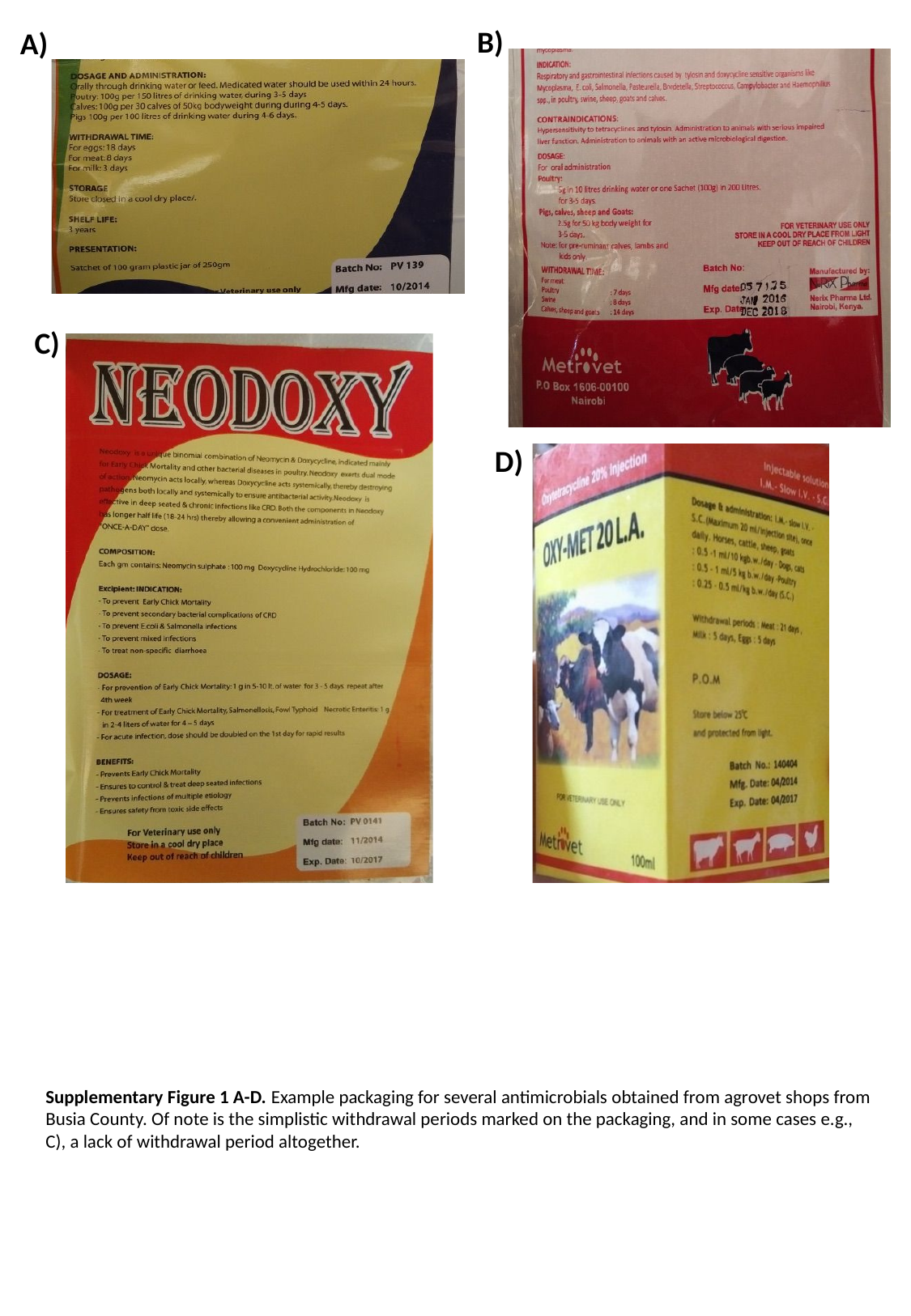

B)
A)
C)
D)
Supplementary Figure 1 A-D. Example packaging for several antimicrobials obtained from agrovet shops from Busia County. Of note is the simplistic withdrawal periods marked on the packaging, and in some cases e.g., C), a lack of withdrawal period altogether.
